# Fermentation design and process optimization strategy based on machine learning

**DOI:** 10.1016/j.bidere.2025.100002

**Published:** 2025-02-26

**Authors:** Zhen-Zhi Wang, Du-Wen Zeng, Yi-Fan Zhu, Ming-Hai Zhou, Akihiko Kondo, Tomohisa Hasunuma, Xin-Qing Zhao

**Affiliations:** a State Key Laboratory of Microbial Metabolism, and School of Life Sciences & Biotechnology, Shanghai Jiao Tong University, Shanghai, 200240, China; b Engineering Biology Research Center, Kobe University, 1-1 Rokkodai, Nada, Kobe, 657-8501, Japan

**Keywords:** Machine learning, Fermentation optimization, Automated fermentation process control, Efficient bioproduction, Process Design

## Abstract

Fermentation optimization is important for industrialization of biological manufacturing, and has been widely applied to diverse sectors including medicine, food, cosmetics and bioenergy, which is related to substantial economic benefits. Strain development is considered to be the core part of fermentation technology, as it directly influences the product yield and overall success of the fermentation process. However, fermentation design and process optimization also play a crucial role in fully exploring the genetic potential of engineered strains for efficient bioproduction. Due to the fact that fermentation process is influenced by complex factors, so far, machine learning has been widely used in this area with its strong capabilities of simulation and prediction. This review provides a brief introduction to the process of fermentation design and process optimization based on machine learning. In the workflow, experimental design strategy is fundamental to explore and characterize the performance of fermentation system. Then, machine learning modelling is employed to simulate the operation of fermentation system and the appropriate fermentation conditions, such as medium composition and process parameters, will be determined. Moreover, in recent years, some extension ideas of fermentation design based on machine learning have also been proposed, including automated fermentation process control, data mining for exploring strain characteristics, transfer learning, hybrid model building, and soft sensor construction. These strategies have created more application scenarios for machine learning, enhancing its adaptability in designing and optimizing the complex fermentation system for efficient bioproduction.

## 1 Introduction

Fermentation is the process by which specific metabolic pathways of microorganisms are utilized to produce metabolites. With the rapid development of industry, modern fermentation technology enables large-scale production of bio-based products. Using renewable substances as raw materials, fermentation technology contributes to sustainable economic growth, which is beneficial to carbon peaking and carbon neutrality goals. Strain development is the key issue of fermentation engineering, but it is also crucial to design and optimize fermentation process from the perspective of systematic optimization to achieve efficient bioproduction.

In the fermentation process, temperature [1,2], pH [3,4], dissolved oxygen [5], nutritional conditions (nitrogen source [6], carbon source [7], mineral salts and vitamins [8]), feeding strategy [9] and other factors will strongly impact the titer and yield of final products (Table 1), which should be optimized in order to fully exploit the production capacity of engineered strains. Due to the complex and dynamic interaction between microorganisms and fermentation environment, applying mechanism models to reflect specific effects of various fermentation conditions and simulate operation of fermentation system poses a great challenge [10]. For example, fermentation kinetics models are mathematical models describing cell growth, substrate utilization and product generation [11]. However, these models often struggle to fully represent the complex internal metabolic processes within organisms and fail to predict the impact of factors beyond substrate concentration. Therefore, empirical models are often utilized to represent the input-output relationships between various factors and fermentation results. Empirical models are black-box models that do not consider the internal mechanisms, and methods of machine learning (ML), a subset of artificial intelligence (AI), are commonly used to construct such models [12].

**Table 1:** Key factors in fermentation process.

Factor	Mechanism	Example
Temperature	Affecting cell growth	[1,2]
	Changing enzyme activity	
	Affecting physical properties of fermentation broth	
pH	Causing physiological stress	[3,4]
	Altering metabolic characteristics	
	Triggering chemical reactions	
Stirring speed and ventilation	Changing dissolved oxygen levels	[5]
	Improving the overall homogeneity to promote the cell's absorption of dissolved oxygen and nutrients	
Nitrogen source	Participating in protein synthesis	[6]
	Changing osmotic pressure and pH	
Carbon source	Providing matter and energy for metabolism	[7]
	Changing osmotic pressure and pH	
Mineral salts and vitamins	Supporting life activities as important cofactors	[8]
Feeding rate	Ensuring appropriate substrate supply	[9]
	Avoiding metabolic adjustments	

ML refers to using empirical data and specific algorithms on computer to generate models with predictive functions [13]. This process is data-driven, which derives statistical relationships among variables from training data. Random Forests (RF) [14], Support Vector Machine (SVM) [15] and Artificial Neural Network (ANN) [16] are typical ML algorithms for simulating and optimizing the fermentation process. By capturing the input-output relationship of fermentation system, these models are able to uncover the complex patterns of interaction and identify optimal fermentation conditions. In addition, ML methods hold great potential for modifying metabolic pathways in strains, which can identify potential overexpression targets for strain engineering [17], screen for the best combination of biological elements [18], guide the fine tuning of enzyme levels [19], facilitate de novo promoter design [20], and predict the impact of 5’ untranslated region on translation efficiency [21].

**Fig. 1:**
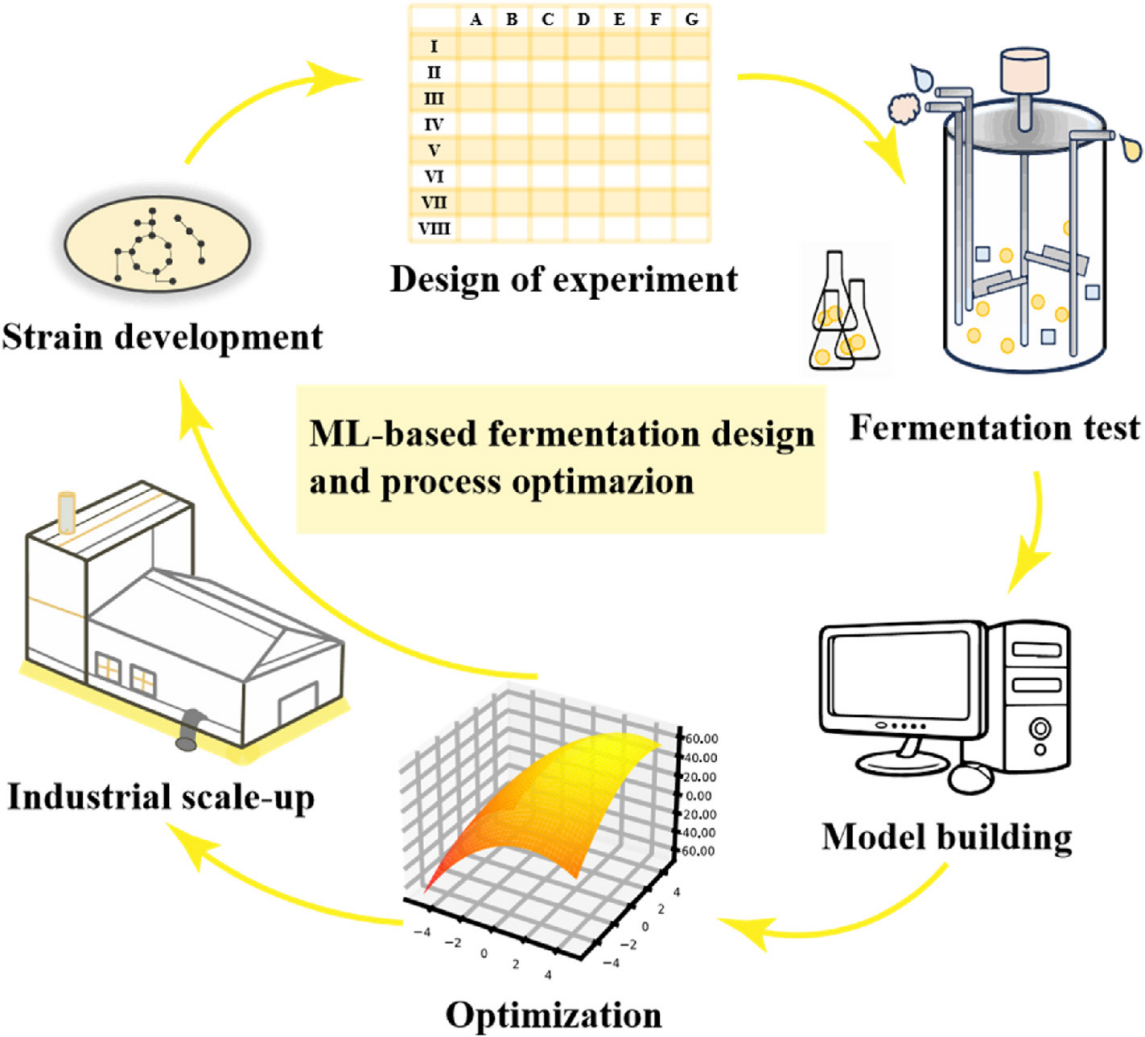
The workflow of ML-based fermentation design and process optimization. Strain development is the first step of fermentation engineering. Then, fermentation test is applied to explore and characterize the performance of fermentation system. By ML modelling and prediction, the appropriate fermentation parameters will be determined. The results of fermentation optimization can be used as reference and basis for strain development. Before industrialization, scaling-up is required to expand the fermentation process that is originally conducted on a laboratory scale to an industrial level.

Today, advancements in synthetic biology have accelerated the speed of strain development and iteration, imposing higher demands on the efficiency of fermentation optimization for newly engineered strains. In the era of AI, high-performance ML algorithms and high-throughput fermentation test equipment have been developed, providing impetus for rapid development in the field of fermentation optimization [22]. Some existing reviews have summarized the progress of ML-based fermentation optimization [[23], [24], [25], [26], [27]]. However, a comprehensive introduction to the overall processes and methodologies involved is still lacking, and there is no centralized categorization of the extended applications of ML methods in this field. To meet these challenges, here we summarize the algorithms and the concrete workflow of ML-based fermentation design and optimization (Fig. 1), comprising experimental design strategy for fermentation test, ML modeling methods, model evaluation, hyperparameter tuning and prediction as well as extension ideas of ML-based fermentation design.

## 2 Experimental design for fermentation test

**Fig. 2:**
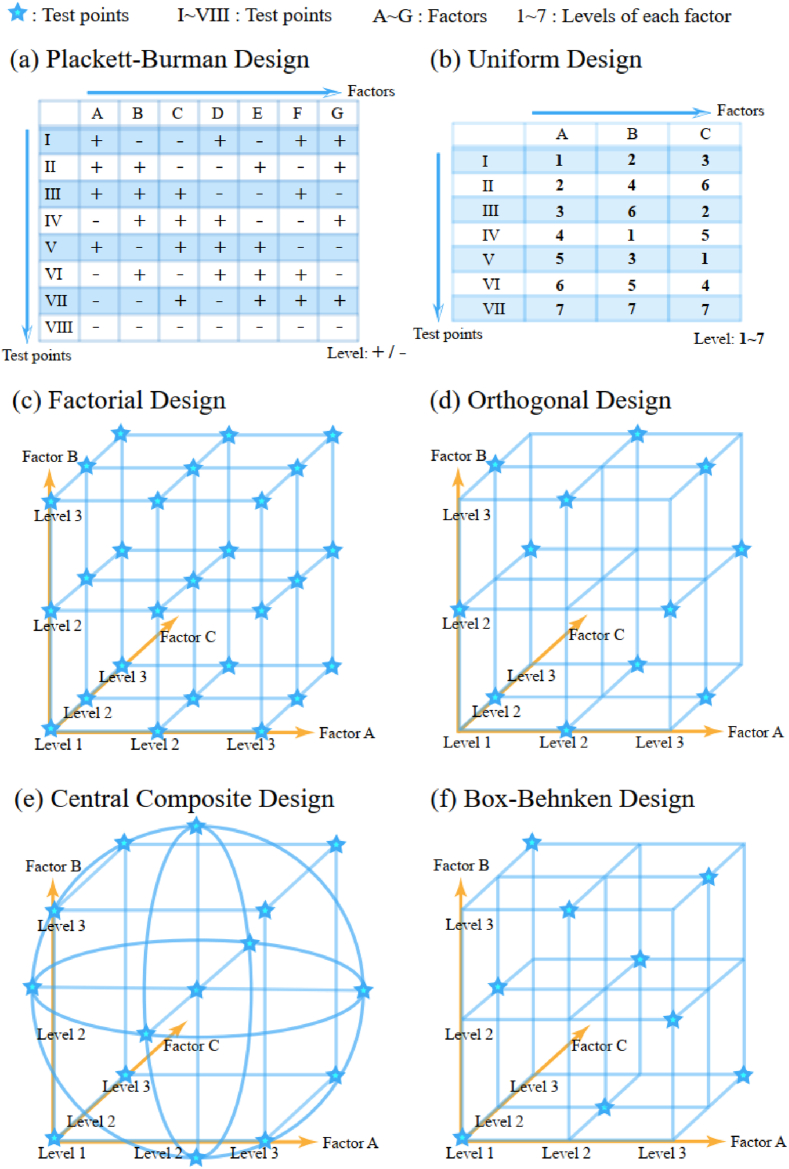
Schematic diagram of DOE (Design of experiment) strategy. DOE is a comprehensive process development strategy, with capacity of evaluating the interactions among various factors. Capital letters represent different factors. Greek numbers in (a)–(b) and pentagram dots in (c)–(f) represent test points. Test point is a single experiment combining different levels of factors.

Fermentation test serves as a cornerstone for investigating and characterizing the performance of fermentation system. So far, One-factor-at-a-time (OFAT) experiments such as removal experiments and supplementation experiments have been widely used [27], despite being time-consuming and yielding unstable results. To enhance the effectiveness of fermentation test, a comprehensive process development strategy has been developed, like DOE (Design of Experiment) strategy based on statistical principles [28], as depicted in Fig. 2, including Factorial Design [29], Plackett-Burman Design [30], Orthogonal Design [31], Uniform Design [32], Central Composite Design [33] and Box-Behnken Design [34]. The task of DOE can be facilitated by relevant software, such as Design Expert - a software tailored for experimental design developed by Stat-Ease, which can generate various experimental design schemes and conduct basic statistical analysis [35].

Compared to DOE strategy, One-factor-at-a-time (OFAT) experiments are appropriate in preliminary screening and determining the optimal level of relatively independent factors [36]. However, the interaction between different factors cannot be ignored. The primary advantage of DOE lies in its capacity to evaluate the interactions among various factors while reducing the number of experiments required. For instance (see Table 2), Plackett-Burman Design is suitable for preliminary screening of various factors and identifying dominant ones, which has been widely used in exploring the effects of mineral salts [37,38]. Orthogonal Design and Uniform Design are used to assess the correlations among various factors with different levels [39,40]. Central Composite Design [38,41] and Box-Behnken Design [42] are specially used for establishing mathematical relationships between variables and outputs.

**Table 2:** DOE strategy and examples.

DOE strategy	Example	Product	Productivity	Reference
Factorial Design	Identifying mineral salts with significant effect	Ethanol	0.42 ​g/g sugar in 12 ​h from industrial wastewater	[8]
Plackett-Burman Design	Studying the effects of supplementation of different mineral salts	Ethanol	50.4 ​g/L after nutritional supplementation, 7.4-fold higher than non-supplemented	[37]
Orthogonal Design	Optimization of temperature, fermentation time, initial pH and liquid loading volume (L_9_[3^4^])	Amylase	20.59 U/ml under optimal conditions, 7-fold higher than initial condition	[39]
Uniform Design	Optimization of glycerol, yeast powder, peptone, KH_2_PO_4_, MgSO_4_, and ZnSO_4_ (U_10_(10^6^))	Ergosterol peroxide	256 ​μg/L, 5-fold higher than initial condition	[40]
Central Composite Design	Identifying the effects of glucose, sodium glutamate and sea salt	Docosahexaenoic acid (DHA)	From 6.18 ​g/L to 8.33 ​g/L after optimization	[38]
Box-Behnken Design	Optimization of initial pH, temperature, moisture content, and incubation period using alfa as sole carbon source	Xylanase and CMCase	826.63 U/gds for xylanase activity, increased by 25 ​%. 61.17 U/gds for CMCase activity, increased by 26 ​%	[42]

In general, when it is not clear which factors have relatively large impacts on the fermentation results and are most worthy of optimization, one can start with an Plackett-Burman Design [38], or a Minimum Run Resolution IV Design [41]. However, these methods cannot quantitatively assess the optimal level of each factor compared to other DOE strategies. Uniform Design requires relatively less workload, but sacrifices a certain degree of accuracy because it does not consider the equal occurrence of different level combinations. In case of three factors each of which are to be tested with three levels, Factorial Design, Central Composite Design, Orthogonal Design and Box-Behnken Design require 27, 15, 12 and 9 experimental tests, respectively (see Fig. 2). With a larger number of test points, the workload will increase, and the evaluation of the impact of various factors and their interactions will be more accuracy. Therefore, DOE strategy should be reasonably selected according to the requirements of accuracy and experimental conditions. The selection of factor levels also significantly influences the validity of the results; thus, it is essential to have a good understanding of the strain's characteristics and to consult relevant publications for guidance. What's more, it is important to note that when testing the influence of certain factors, it is necessary to keep other factors at an appropriate level based on experiences.

Based on DOE, traditional statistical analysis methods such as ANOVA can be employed to preliminarily assess the impact of various factors and directly select the best level, but the construction of ML models can yield more precise predictive results. There is no strict correspondence between specific ML algorithm and particular experimental design strategy. Theoretically, different experimental design strategy may be suitable for different ML algorithms. However, multiple ML algorithms can be trained on the same set of data, and the best performing model can be selected afterwards [43]. Data obtained from Factorial Design, Orthogonal Design, Uniform Design, and Response Surface Methodology can all be utilized for ML model training. Furthermore, we can conduct a new batch of experiments based on previous experiment, and put them together for model construction. A typical example is constructing a new Central Composite Design experiment originating from Factorial Design [8].

## 3 Modelling methods for fermentation optimization

### 3.1 Overview

After obtaining fermentation data, ML algorithms can be employed for model building to predict the fermentation results under various conditions. This necessitates a certain level of proficiency in mathematical statistics and computer technology. In the age of AI, powerful tools have emerged, which drastically lower the barrier of usage and facilitate widespread application of ML across diverse research fields. Python, as one of the most popular programming languages for ML, has powerful packages such as Scikit-learn [44,45]. MATLAB also has a regression model toolbox containing various ML algorithms [46].

In addition to the algorithm, data quality also represents a critical factor that impacts the accuracy of the model. Thus, the fermentation data requires preprocessing before model training [47]. For example, each feature of data, such as sugar input (g/L) and temperature (°C), has different value range, variance, and unit of measurement. By standardization or normalization, the preprocessed data preserves original characteristics, eliminates inconsistency and enhance model reliability. In the workflow of ML-based fermentation design and process optimization depicted in Fig. 1, variables involved in fermentation tests are limited and hypothetically influential, so the quality of data is only affected by experimental design and the accuracy of the fermentation result detection. However, if we acquire data from fermentor or even extract information from the literature, as presented in section 5 (**Extension ideas of ML-based fermentation design**), there may be some missing data or outliers in the dataset. Then, missing value handling and data cleaning should be applied to enhance data quality.

### 3.2 Response surface methodology

Response Surface Methodology (RSM) is not a ML method in the traditional sense, but it also attempts to find the relationship between input variables and output variables. RSM is a simple and effective modelling method which can be completed by statistical analysis software [48]. Based on the steepness and shape of response surface, it is possible to observe the influence of each factor and the interactions. With regression equation, the optimal fermentation conditions can be predicted [38,41,42,49]. However, the model parameters constructed by RSM are relatively insufficient. A second-order model only includes the first and quadratic term (x, x^2^) of each factor, as well as the interaction term between two factors (x_1_x_2_), which lacks the ability to characterize the complex nonlinear interactions in fermentation system [48].

### 3.3 Typical ML algorithms

Decision Tree is a ML method based on tree structure [50]. This model can only predict the approximate range of fermentation result [51]. Random Forests (RF) is an integration algorithm of decision tree with improved accuracy [14]. By calculating the average of the predictions from all trees rather than taking the majority vote, Random Forests Regression (RFR) is capable of addressing regression tasks for fermentation optimization [52]. The advantages of RFR include rapid training speed, anti-interference capability and proficiency in handling high-dimensional data and capturing non-linear relationships. However, RFR is inevitably influenced by decision tree partitioning boundaries, and the predicted fermentation curve presents a discontinuous ladder shape, which is not suitable for describing the process of fermentation results changing over time [53].

Support Vector Machine (SVM) is a popular ML algorithm that uses hyperplanes to partition the sample space and achieve classification of sample data [15]. The final outputs in SVM are heavily influenced by a few key samples, indicating that the algorithm possesses not only simplicity but also robustness. In contrast, Support Vector Regression (SVR) is a regression algorithm specifically designed to identify hyperplanes that optimally fit the data [54]. Experimental results indicate that SVR possesses robust fitting and prediction capabilities especially for a small amount of data [55]. Therefore, if obtaining sufficient fermentation data is difficult due to limited experimental condition, SVR can be used for data fitting and analysis.

### 3.4 Deep learning and other ML methods

Since 2012, deep learning based on neural networks has experienced a rapid expansion, with Backpropagation-Artificial Neural Network (BP-ANN) gaining popularity in the field of fermentation optimization [53,[56], [57], [58]]. BP-ANN simulates the synaptic structure of the brain with multiple neurons to process information [16]. Each neuron receives input signals from other neurons, combines them based on the weights assigned to each input neuron, and then produces an output signal through an activation function. In comparison to RSM, BP-ANN demonstrates superior prediction accuracy, and can theoretically capture almost any form of the nonlinear relationship. Due to its unique gradient descent training method and large number of parameters, BP-ANN has higher robustness than general ML algorithms.

Apart from RF, SVM and BP-ANN, other ML methods have also been used in fermentation optimization (see examples in Table 3), like Gradient Boosting Machine (GBM) [59], Adaptive Boosting (AdaBoost) [59], eXtreme Gradient Boosting (XGBoost) [60,61], Gaussian Process Regression (GPR) [43], Bayesian Optimization [62], Functional Link Artificial Neural Network (FLANN) [63] and Least-Squares Support Vector Machine (LS-SVM) [64,65]. The development of new ML algorithms is always an ongoing process, but the No Free Lunch theorem states that there is no universal learning algorithm that performs well on all kinds of tasks [66]. In fact, some algorithms frequently perform better than others in certain cases. Thus, the understanding of the applicability of different ML algorithms is beneficial for researchers.

**Table 3:** Modelling methods and examples. RSM: Response Surface Methodology. DT: Decision Tree. RFR: Random Forests Regression. SVR: Support Vector Machine Regression. BP-ANN: Backpropagation-Artificial Neural Network. GBM: Gradient Boosting Machine. AdaBoost: Adaptive Boosting. XGBoost: eXtreme Gradient Boosting. GPR: Gaussian Process Regression. BO: Bayesian Optimization. FLANN: Functional Link Artificial Neural Network. LS-SVM: Least-Squares Support Vector Machine.

Modelling methods	Example	Product	Result or conclusion	Reference
RSM	Searching for optimized conditions of temperature, glucose, biotin and urea concentration	Glutamic acid	Experimental value (19.9 ​mg/mL) was quite close to predicted value (19.84 ​mg/mL)	[49]
RSM	Identifying the effects of glucose, sodium glutamate and sea salt	Docosahexaenoic acid	Titer was increased from 6.18 ​g/L to 8.33 ​g/L after optimization	[38]
RSM	Optimization of initial pH, temperature, moisture content, and incubation period	Xylanase and CMCase	Xylanase activity was increased by 25 ​% (826.63 U/gds) and CMCase activity was increased by 26 ​% (61.17 U/gds)	[42]
DT	Predicting high yield conditions for lipid production using *Y. lipolytica*:	Lipid	Features that lead to high biomass production and lipid content were successfully identified	[51]
RFR	Predicting ethanol production curve in mixed-sugar fermentation	Ethanol	RFR leads to “stair-like” lines of fermentation curve	[53]
SVR	Microbial lipid production from cellulosic ethanol wastewater	Lipid	SVR performs better than BP-ANN especially with few samples	[55]
BP-ANN	Predicting ethanol production curve in mixed-sugar fermentation	Ethanol	BP-ANN demonstrates potential for application in transfer learning;	[53]
			The robustness of BP-ANN surpasses that of SVR and RFR	
BP-AN	Optimization of Brewers' spent grain, yeast extract and salt solution concentration	Poly-β hydroxybutyrate	Experimental value (916 ​mg/L) was very close to predicted value (916.31 ​mg/L)	[56]
BP-ANN	Pretreatment process parameters optimization for total reducing sugar production	Reducing sugar	The predicted value of BP-ANN was more accurate than RSM	[57]
BP-ANN	Optimization of residual sugar (RS), AN, DO, and C/N ratio	L-lysine	BP-ANN can be easily combined with GA and NSGA-II for multi-objective optimization	[58]
GBM	Modelling H_2_ production from wastewater by dark fermentation process	H_2_	The performance of GBM in test data was similar to SVR and RFR	[59]
AdaBoost	Modelling H_2_ production from wastewater by dark fermentation process	H_2_	AdaBoost performed slightly worse than SVR, GBM, and RFR	[59]
XGBoost	Optimization of 11 process conditions for maximum caproic acid	Caproic acid	XGBoost performed significantly better than RFR, ANN and SVR	[60]
GPR	Predicting biomass production from predictors obtained by data mining	Biomass	GPR model reached the highest predicting accuracy compared with the other 25 models	[43]
BO	Optimization of concentrations of carbon sources, nitrogen sources and salts	S-Adenosyl-L-methionine (SAM)	SAM titer increased 91.6 ​% than unoptimized medium, and increased 28.2 ​% than medium optimized by orthogonal tests	[62]
FLANN	Predicting continuous biohydrogen production from 12 input variables	H_2_	FLANN can handle complex data with many variables	[63]
LS-SVM	Optimization of process parameters for anaerobic fermentation	Cumulative biogas	Biogas production was 14.13 ​% higher than that of optimal conditions in orthogonal experiment	[64]

## 4 Evaluation, hyperparameter tuning and optimal parameters screening

### 4.1 Evaluation and hyperparameter tuning

Error is the discrepancy between model prediction and the actual result, which is utilized to assess the model's capability of fitting and generalization. Mean Square Error (MSE), Root Mean Square Error (RMSE), and Correlation Index (R^2^) are commonly employed for model evaluation. Different algorithms possess distinct characteristics and perform well in different tasks, so researchers often train several models on the same set of data in order to identify the best algorithm [43].

ML models have hyperparameters that determines the model structure as well as the training procedure, which we must set to customize the model to specific data [67]. By carefully adjusting hyperparameters, we will help models to learn universal rules that can be applied to more potential samples. Tuning hyperparameters manually requires a high level of expertise from researchers, so automatic hyperparameter tuning methods, such as Grid Search, Random Search, Bayesian Algorithm and Genetic Algorithm have been developed. When dealing with models containing few hyperparameters and only running for a short time, complex algorithms are often unnecessary. Given that ML models used in fermentation optimization do not possess a certain level of complexity and can be finished within minutes, Huang chose to optimize hyperparameters for RFR, SVR, and BP-ANN models using Grid Search method to evaluate all combinations of hyperparameters [53].

### 4.2 Determination of optimum parameters

The main target of incorporating ML models into fermentation process is to identify the optimum parameters, or the combination of factors. By leveraging ML models to determine the best parameters, higher production efficiency can be achieved. While traditional fermentation optimization focuses on a single production indicator such as titer, we advocate for the necessity of Multi-Objective Optimization (MOO) to maximize economic benefits. In MMO, a series of objectives are optimized simultaneously based on different levels of importance. For instance, the objectives could be maximizing the product formation rate and the substrate consumption rate, while maintaining a moderate cell growth rate [58]. For fed-batch fermentation process, the objectives could be maximizing the total output of products while minimizing the amount of feed, to prevent the waste of raw materials [68]. The common algorithms for MOO include Genetic Algorithm and NSGA-II [58].

**Fig. 3:**
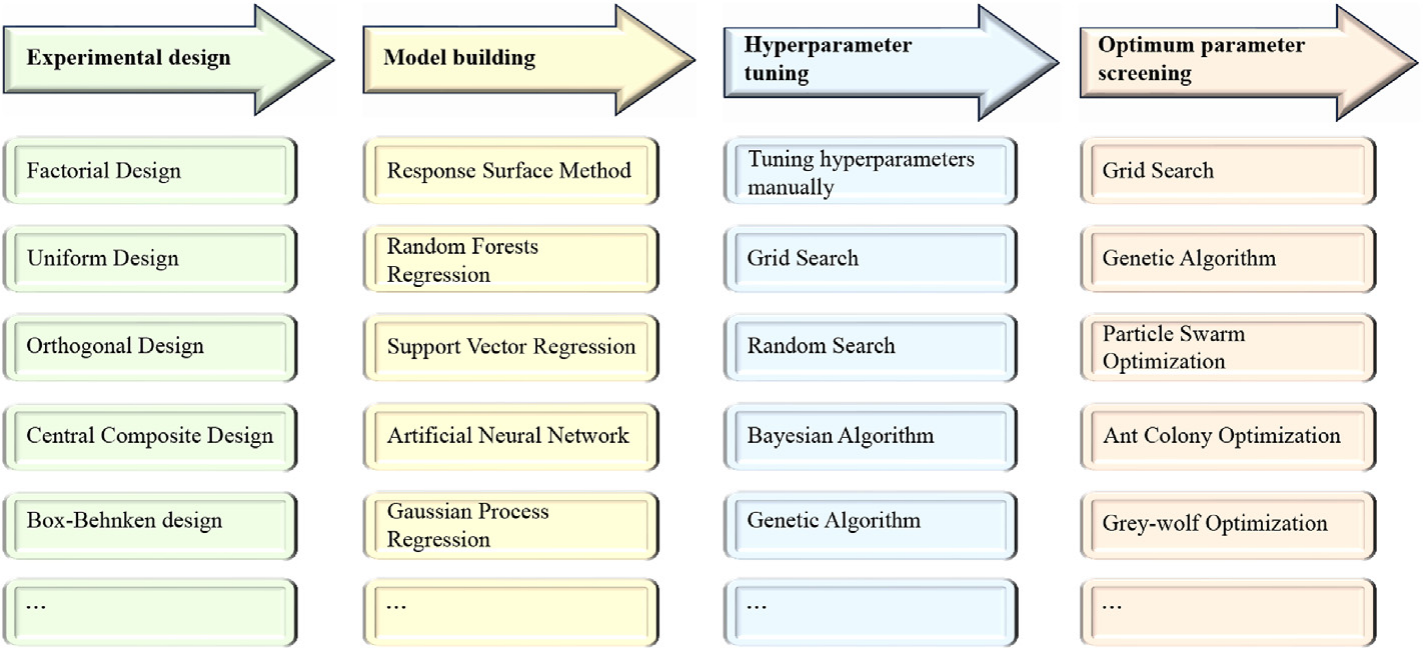
Methodology of ML-based optimization. The methodology for determining optimal parameters and hyperparameters in ML models shares commonalities. For example, Grid search is applicable for both hyperparameter tuning and optimum fermentation parameter screening.

The methodology for determining optimal parameters and hyperparameters in ML models shares commonalities. Grid Search refers to exploring a specified hyperparameter space completely to select optimal hyperparameters. It has been used for determining the optimal pretreatment conditions for biogas fermentation from corn straw [62]. Genetic algorithm (GA) is a popular optimization algorithm inspired by the principles of natural selection [69]. It can be easily integrated with other ML algorithms, such as SVM [55] and BP-ANN [58,70]. Other intelligent optimization algorithms similar to Genetic Algorithm like Particle Swarm Optimization (PSO) [63,71], Grey-Wolf Optimization (GWO) [65] have also been used in determining optimum parameters. In the previous text, different algorithms and methods are mentioned and their relationship is shown in Fig. 3.

## 5 Extension ideas of ML-based fermentation design

### 5.1 Automated fermentation process control

**Fig. 4:**
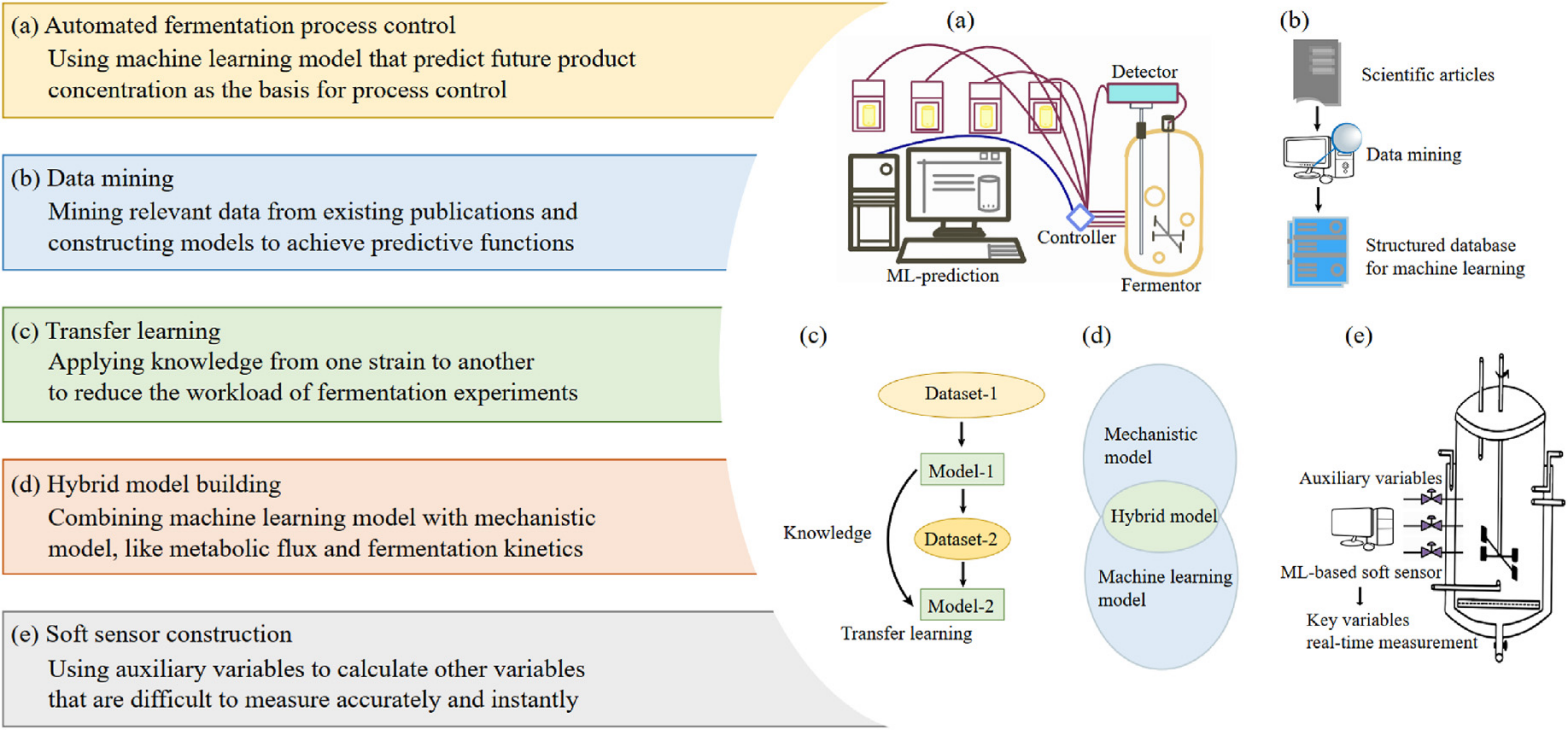
Extension ideas of ML-based fermentation design and process optimization. In recent years, new strategies such as automatic control, data mining, transfer learning, hybrid model building and soft sensor construction have enhanced the adaptability of ML in designing and optimizing the complex fermentation system, which represent the future direction of this field.

Fermentation process control is another aspect of fermentation design and process optimization (Fig. 4a). For example, the feeding rate and time in batch fermentation have a strong impact on the fermentation system. Improper feeding strategy can result in starvation, byproduct accumulation, substrate waste and other issues. Huang et al. developed an automatic control system for batch fermentation [9], which utilizes an ANN model to predict glycerol concentration and product concentration 60 ​min later, with the initial glycerol concentration, the added volume of glycerol/NaOH and other data as input variables. The system also automatically adjusts the feeding rate to maintain the glycerol concentration at a low level. This automated system not only eliminates the need for real-time detection and manual control but also effectively increases 1,3-propanediol production of *C. butyricum* while reducing substrate waste. In addition to the feeding strategy, other process parameters can be controlled automatically as well. In Mesquita's work, air flow rate was effectively controlled using an ANN-based micro-aerobic fermentation strategy [72]. The model offered setpoints for the controller, enabling the adjustment of inlet air flow to regulate oxygen uptake. This strategy provided a volumetric ethanol productivity of 4.16 ​g ​L^−1^ ​h^−1^ and a yield of 0.48 g_ethanol_.g_substrate_^−1^, which were higher than other studies. Apart from ANN, Least-Square Support Vector Machine (LS-SVM) has also been used to predict future product concentration [65]. Reinforcement learning can also help to solve the control problem of fed-batch fermentation. By iteratively designing experiments and learning the influence of the feeding rate on product accumulation, a reinforcement learning model capable of predicting the best control scheme can be developed [68].

### 5.2 Data mining for exploring strain characteristics

ML is data-driven. After mining relevant data information from existing publications, models can be constructed to achieve certain predictive functions, providing reference for fermentation optimization (Fig. 4b). Pensupa et al. conducted data mining on *Y. lipolytica* and organized it into a database containing 15 publications [43]. Multiple regression models were used to accurately predict biomass, with the best model achieving an R^2^ value of 0.90. Similar works have been done by Coşgun. Using a dataset containing 356 instances from 22 publications [51]. Through decision trees, most influential features that lead to high biomass production, lipid content and lipid production are identified. In order to save manpower and improve efficiency, Xiao et al. used the natural language processing tool GPT-4 to extract information from articles about *Y. lipolytica* for model training [73]. Random Forests algorithm showed the best performance and predicted the fermentation titers with high accuracy, reaching an R^2^ value of 0.86 in test data. In conclusion, data mining tells us some valuable information about the characteristics of strains. However, the diversity and heterogeneity of searched data contribute to limited prediction accuracy, making it difficult to be applicated in a specific research of fermentation design and process optimization.

### 5.3 Transfer learning

Transfer learning refers to applying knowledge from one task to another, to enable the adaptation of models trained on large datasets to new, possibly smaller or more specialized datasets [74] (Fig. 4c). It is a new breakthrough with the purpose of reducing the number of experiments. Engineered strains with similar physiological characteristics share some commons during the fermentation process, which is the foundation of transfer learning. If an existing model has identified common features between two systems, it is theoretically possible to be applied to another system with a small amount of additional data. Huang et al. utilized ANN for transfer learning in mixed-sugar fermentation [53] and ACSE pretreatment optimization [75]. The *Y. lipolytica* model constructed by Xiao et al. also achieved transfer learning on *R. toruloides*, successfully predicting the impact of gene manipulation on astaxanthin production [73]. However, under normal circumstances, newly engineered strains often exhibit great changes in metabolic characteristics, posing challenges in utilizing the fermentation model of one existing strain for transfer learning into another strain. One potential strategy for enhancing transfer learning performance is to modularize the transfer model and separate domain-specific information from generalizable information [74]. This can be achieved by employing hybrid models, where a kinetic model simulates known knowledge and a data-driven model simulates the unknown part.

### 5.4 Hybrid model building

The mechanism model is a simulation of the composition and operation of fermentation system, including intracellular metabolic flux and fermentation kinetics. With the provided initial conditions, these models are capable of predicting the performance of fermentation system. However, a single mechanism model is difficult to describe the entire fermentation process both inside and outside the cell due to the lack of knowledge, so a hybrid model is constructed with ML as a viable complement [76] (Fig. 4d). For example, omics data reflecting the intrinsic characteristics of strains is now generated at an unprecedented rate but is hard to be interpreted [77]. Culley's work showed that metabolic flux omics data and transcriptomics data of *S. cerevisiae* can be used together for ML modelling in growth rate prediction [78]. In a previous review, Wan et al. systematically introduced how omics data are utilized in process optimization by determining the cell needs during the fermentation process [79]. However, so far, the applications of omics data in fermentation engineering are still mainly limited to mechanism analysis for strain development [[80], [81], [82]].

Another example is the combination of ML models and fermentation kinetics. At the system level, conventional kinetic model is not accurate enough in predicting fermentation process. However, fermentation kinetics can be used to extract features contained in the fermentation system and then generate data for ML, making it more robust to the interference of noise. Lai et al. used generated data from kinetic equations as input to ML models for predicting three off-line parameters and achieved higher predictive power [83]. Even the parameters of kinetic model can be estimated with the help of ML. Ismail et al. proposed that Artificial Bee Colony and other optimization algorithm can be used for fitting the simulated model into the experimental data to obtain more accurate values [84].

In short, integrating ML models with metabolic models may be the future solution. Through the simulation of genotype-phenotype-environment relationships, the reliance on fermentation test data could be authentically reduced.

### 5.5 ML-based soft sensor construction

Accurate real-time measurement of key variables is essential for effective monitoring and control of the fermentation process [85]. Apart from traditional fermentation sensor, soft sensor has become a new trend. Soft sensor technology refers to using simple and precise auxiliary variables to calculate other variables that are difficult to measure accurately and instantly, which is a data-driven process (Fig. 4e). For example, colors of pH indicator array were used for determination of ethanol content [86]. Volatile composition of lignocellulosic biomass was used for estimating cell growth and ethanol production [87]. In comparison, more auxiliary variables were used by Hua to predict penicillin concentration [88]. Spectroscopic data collected with ATR-MIR was also used for real-time state estimation based on Partial Least Squares Regression, then a hybrid model with the kinetic model was constructed and obtained better performance [89]. Certainly, the feature selection of high-dimensional auxiliary variables is crucial for the development of soft sensor, and their feature importance must be adjusted before model training [90]. Another way to improve the robustness of soft sensors is to utilize transfer learning strategy using data from multiple operating conditions in different fermentation batches [91]. In conclusion, soft sensor eliminates the reliance on offline detection, such as HPLC, allowing us to easily obtain large amounts of fermentation data. By utilizing these data, it is possible to model the operation of fermentor more accurately, which could potentially lead to a qualitative leap in feeding strategy and process control strategy.

Moreover, it is worth noting that biosensors, which are widely used for screening of high-yield strains, are also potential tools for fermentation process monitoring. Examples include FRET-based lysine biosensor [92], transcription activator protein-based xylose biosensor [93], transcription factor-based adipic acid biosensor [94] and glycolate biosensor [95]. For the detection of a single product, biosensors can achieve lower detection costs and higher throughput than HPLC. However, the fundamental problem with biosensors is that the detection of metabolites is not real-time, but requires a certain reaction time.

## 6 Discussion and future prospects

Fermentation design and process optimization are crucial for exerting the production performance of engineered strains, and providing important references for the iterative development of strains. In this review, we showed how ML methods play a role in the field of fermentation engineering, which help researchers to solve statistical analysis problems in fermentation experiments. The main advantage of ML methods over traditional data analysis methods is that ML can predict the optimal conditions for obtaining the best fermentation results, rather than directly selecting the level of each factor. The application of ML modelling also aims to reduce the number of experiments and further cut down the cost, because the optimal conditions predicted by ML models are more effective with the same amount of data. However, it is important to note that ML is inherently data-driven. There exists a trade-off between enhancing the accuracy of prediction and reducing the number of experiments. What we can do is to carefully design fermentation experiment by determining which factors are worth studying, selecting the appropriate range of values, and deciding which DOE strategy will be used. Meanwhile, we can concurrently utilize multiple ML algorithms for model training on the same dataset.

In addition to the dependence on the amount of data, another challenge of ML methods is the problem of interpretability. Compared to simple models such as linear regression and fermentation kinetics, the decision-making and prediction processes of ML models are difficult to understand. For fermentation process optimization, if the influence of factors and their interactions in ML models can be interpreted, we can gain deeper insights into the underlying mechanisms, as well as assess the reliability of the models. Feature importance analysis and visualization analysis are effective ways to help us analyze the effects of different factors on the results [60]. However, the interpretation of the underlying mechanisms still relies heavily on the experience and expertise of researchers, which limits the application of ML methods in fermentation process optimization.

The problem of scaling up in fermentation optimization and system design also needs consideration. With different environmental heterogeneity of temperature, oxygen and nutrients, the performance of engineered strain may go backwards. Due to limitations in experimental conditions, ML methods are mostly limited to shake flask culture and less commonly used for optimizing fermentor system. The optimized fermentation conditions obtained in shake flask culture is likely to be inappropriate for industrial production. Therefore, further advancements in fermentation optimization are contingent upon the modernization of fermentation test equipment. Xia et al. provided a comprehensive overview of high-throughput parallel reactors and automation technologies [22]. The relatively small-scale parallel reactors closely simulate the industrial fermentation environment, and is sufficiently miniaturized to ensure experimental throughput. On the other hand, automation technology in the entire procedure enables automatic cultivation and detection, enhancing the efficiency of performance validation and fermentation optimization of new engineered strain. Furthermore, Computational Fluid Dynamic (CFD) model serves as a potential tool for addressing scale-up challenges in fermentation [23]. By integrating biological models with CFD models, researchers can anticipate flow field information within fermentor. The simulation results from CFD will offer crucial insights for adjusting fermentation parameters.

In future research, it is necessary to leverage the efficiency of ML in utilizing existing data. What we need to constantly consider is what data can be used to build ML models and what result can be predicted. Specifically, data derived from omics analysis, published literature, and historical fermentation process hold great potential. These data can be obtained in large quantities and used for model training. Omics data reflect the metabolic characteristics of strains, from which we may extract feature that closely related to the metabolic process of product accumulation, as another optimization objective of ML algorithms, or even predict appropriate fermentation conditions directly. By mining information from published literature, we can preliminarily ascertain which factors may have great influence on the growth and metabolism of a specific strain. This will be an important reference for the design of fermentation experiment. Moreover, historical fermentation data also contains a lot of information. Utilizing these datasets allows ML models to learn and elucidate the real-time response characteristics of strains to variations in fermentation parameters. In conclusion, how to develop ML models and make good use of various data have become an important challenge in the field of fermentation design and process optimization.

What needs to point out is that fermentation process design should be closely performed together with strain development. On the one hand, the optimal fermentation process relies on the optimal genetic background of the strain. On the other hand, strain-specific fermentation optimization is important for improved bioproduction efficiency because different natural strains belonging to the same species may have specific metabolic properties, and the same is true for the genetically engineered strains from the same parent strain. Therefore, care should be taken when data from different strains are used for establishing the ML model. In the future, general strategies of fermentation design that are suitable for various strains may be developed, and can be fine-tuned to adapt to different strains. Compared to general ML methods, deep learning is suitable for processing high dimensional and complex data. With the development of automation instruments and accumulation of high-quality data, deep learning will become a more powerful tool for the advancement of smart fermentation design.

## Author contributions

Z. W. drafted the manuscript and designed the figures. X. Z., T. H., and A. K. have read, edited and reviewed the content. D. Z, Y. Z. and M. Z. provided critical feedback for the refinement of the manuscript.

## Funding

We are thankful for the financial support from the State Key Research and Development Program of China (No. 2022YFE0108500), and 10.13039/501100001809National Natural Science Foundation of China (No. 21978168).

## Declaration of competing interest

The authors declare that they have no known competing financial interests or personal relationships that could have appeared to influence the work reported in this paper.
